# Biomechanical properties of five different currently used implants for open-wedge high tibial osteotomy

**DOI:** 10.1186/s40634-015-0030-4

**Published:** 2015-06-18

**Authors:** Arnaud Diffo Kaze, Stefan Maas, Danièle Waldmann, Andreas Zilian, Klaus Dueck, Dietrich Pape

**Affiliations:** Faculty of Science, Technology and Communication, University of Luxembourg, 6, rue R. Coudenhove-Kalergi, L-1359 Luxembourg, Luxembourg; Department of Orthopedic Surgery, Centre Hospitalier de Luxembourg, L-1460 Luxembourg, Luxembourg; Sports Medicine Research Laboratory, Public Research Centre for Health, Luxembourg, Centre Médical de la Fondation Norbert Metz, 76, rue d’Eich, L-1460 Luxembourg, Luxembourg

**Keywords:** High tibial osteotomy (HTO), Osteoarthritis, Biomechanics, Mechanical test, Cyclic and static loading, TomoFix, ContourLock, PEEKPower, iBalance, Stiffness, Valgus malrotation

## Abstract

**Background:**

As several new tibial osteotomy plates recently appeared on the market, the aim of the present study was to compare mechanical static and fatigue strength of three newly designed plates with gold standard plates for the treatment of medial knee joint osteoarthritis.

**Methods:**

Sixteen fourth-generation tibial bone composites underwent a medial open-wedge high tibial osteotomy (HTO) according to standard techniques, using five TomoFix standard plates, five PEEKPower plates and six iBalance implants. Static compression load to failure and load-controlled cyclic fatigue failure tests were performed. Forces, horizontal and vertical displacements were measured; rotational permanent plastic deformations, maximal displacement ranges in the hysteresis loops of the cyclic loading responses and dynamic stiffness were determined.

**Results:**

Static compression load to failure tests revealed that all plates showed sufficient stability up to 2400 N without any signs of opposite cortex fracture, which occurred above this load in all constructs at different load levels. During the fatigue failure tests, screw breakage in the iBalance group and opposite cortex fractures in all constructs occurred only under physiological loading conditions (<2400 N). The highest fatigue strength in terms of maximal load and number of cycles performed prior to failure was observed for the ContourLock group followed by the iBalance implants, the TomoFix standard (std) and small stature (sm) plates. The PEEKPower group showed the lowest fatigue strength.

**Conclusions:**

All plates showed sufficient stability under static loading. Compared to the TomoFix and the PEEKPower plates, the ContourLock plate and iBalance implant showed a higher mechanical fatigue strength during cyclic fatigue testing. These data suggest that both mechanical static and fatigue strength increase with a wider proximal T-shaped plate design together with diverging proximal screws as used in the ContourLock plate or a closed-wedge construction as in the iBalance design. Mechanical strength of the bone-implant constructs decreases with a narrow T-shaped proximal end design and converging proximal screws (TomoFix) or a short vertical plate design (PEEKPower Plate). Whenever high mechanical strength is required, a ContourLock or iBalance plate should be selected.

## Background

High tibial osteotomy (HTO) is a well-established method for the treatment of unicompartmental varus gonarthrosis. Both lateral closing and medial opening techniques exist to produce a valgus alignment (Staubli 2008; Staubli and Jacob 2010). The latter is becoming increasingly popular when performed in a biplanar fashion. Since the introduction of long and rigid-angle stable plates, the frequency of non-unions after an open-wedge HTO has declined significantly (Staubli and Jacob 2010). Apart from a good vascularization of the bone, solid plate fixation is mandatory for rapid bone healing (Staubli 2008; Staubli and Jacob 2010). Although clinical results after HTO often are encouraging some factors associated with a poor long-term outcome such as imprecise osteotomy or loss of the primary correction angle due to poor primary fixation stability of the implant (Spahn et al. 2006; Pornrattanamaneewong et al. 2012).

There are many different implants from different manufacturers available. Biomechanical (Luo et al. 2013; Spahn and Wittig 2002; Stoffel et al. 2004; Zhim et al. 2005; Agneskirchner et al. 2006; Maas et al. 2013; Watanabe et al. 2014) and clinical (Pape et al. 2011; Saeed and Rae 2009; Valkering et al. 2009; Cotic et al. 2014; Woon-Hwa et al. 2013) comparative studies are often performed to help surgeons choose the most appropriate fixation device for the osteotomy.

TomoFix, the long and rigid T-shaped titanium internal fixator with uniaxial locking system (Synthes GmbH, Oberdorf, Switzerland), is currently the gold standard. It combines biomechanical properties that are held accountable for fast bone healing: (1) high primary fixation stability; (2) a compliant bone-implant construct which allows residual micromotion within the osteotomy gap to promote a “callus massage”. TomoFix plates exist in two versions, such as the small stature (sm) version designed for lighter patients and the standard (std) version without weight restriction. Both plates have a narrow proximal design which allows for a biplanar osteotomy, thus enlarging the surface for rapid contact healing (Pape et al. 2010, 2011).

PEEKPower (carbon-fiber reinforced polyetheretherketone (PEEK)) and iBalance (non-absorbable polyetheretherketone)–two implants also indicated for a biplanar osteotomy–were recently designed by Arthrex, a company based in Munich (Germany). The PEEKPower plate is T-shaped, shorter than the TomoFix but coming with a multidirectional locking system. The iBalance implant, a PEEK spacer, is inserted into the osteotomy gap in a uniplanar and closed-wedge-like technique with immediate close contact between the PEEK material and the proximal and distal cortical and spongious bone surface (Pape et al. 2012).

**Fig. 1:**
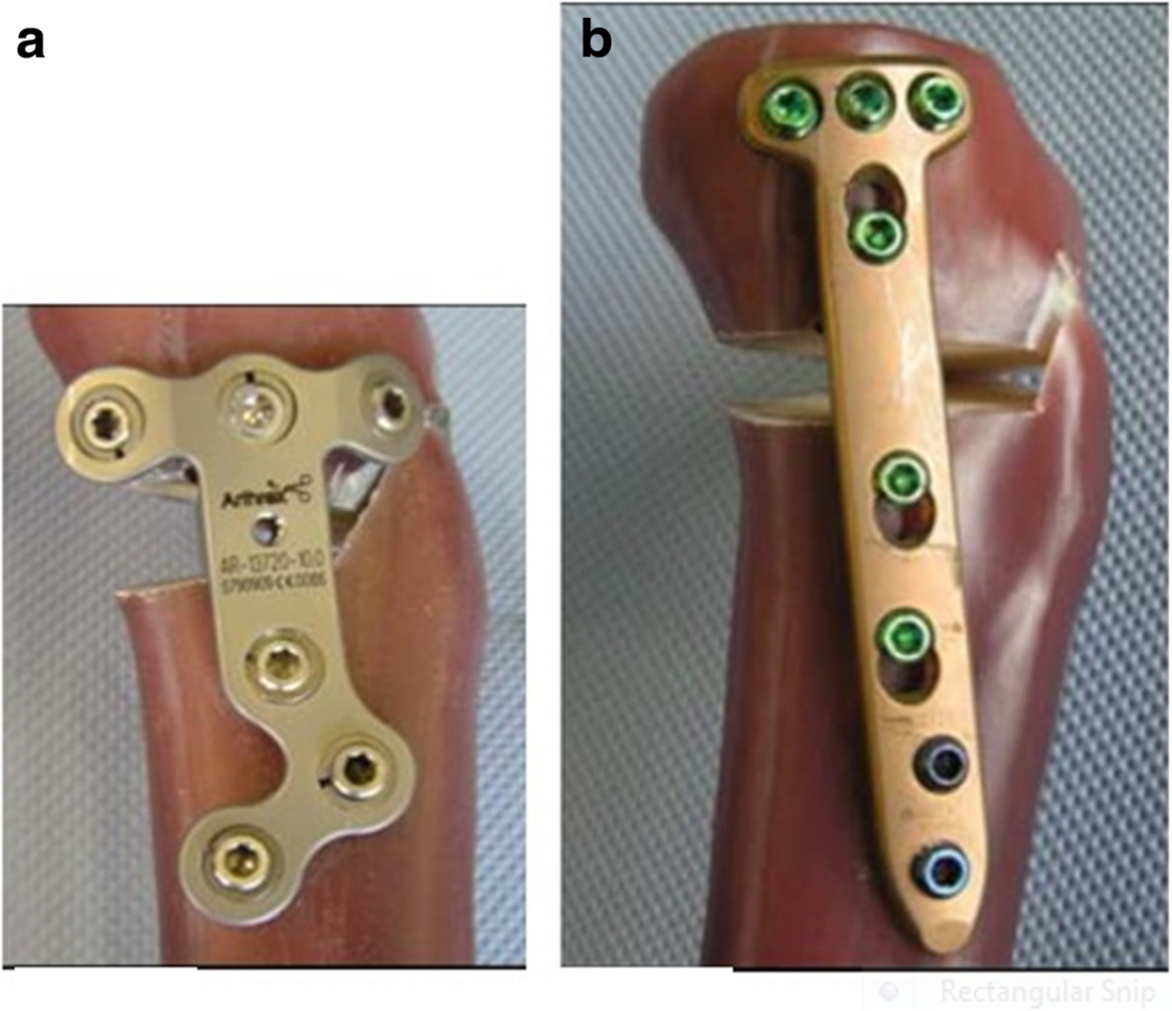
Fixation devices compared by Maas et al. (Maas et al. 2013). (**a**) The ContourLock HTO plate is applicable to patients without weight limitation. (**b**) The TomoFix sm plate is indicated only for small stature patients

**Fig. 2:**
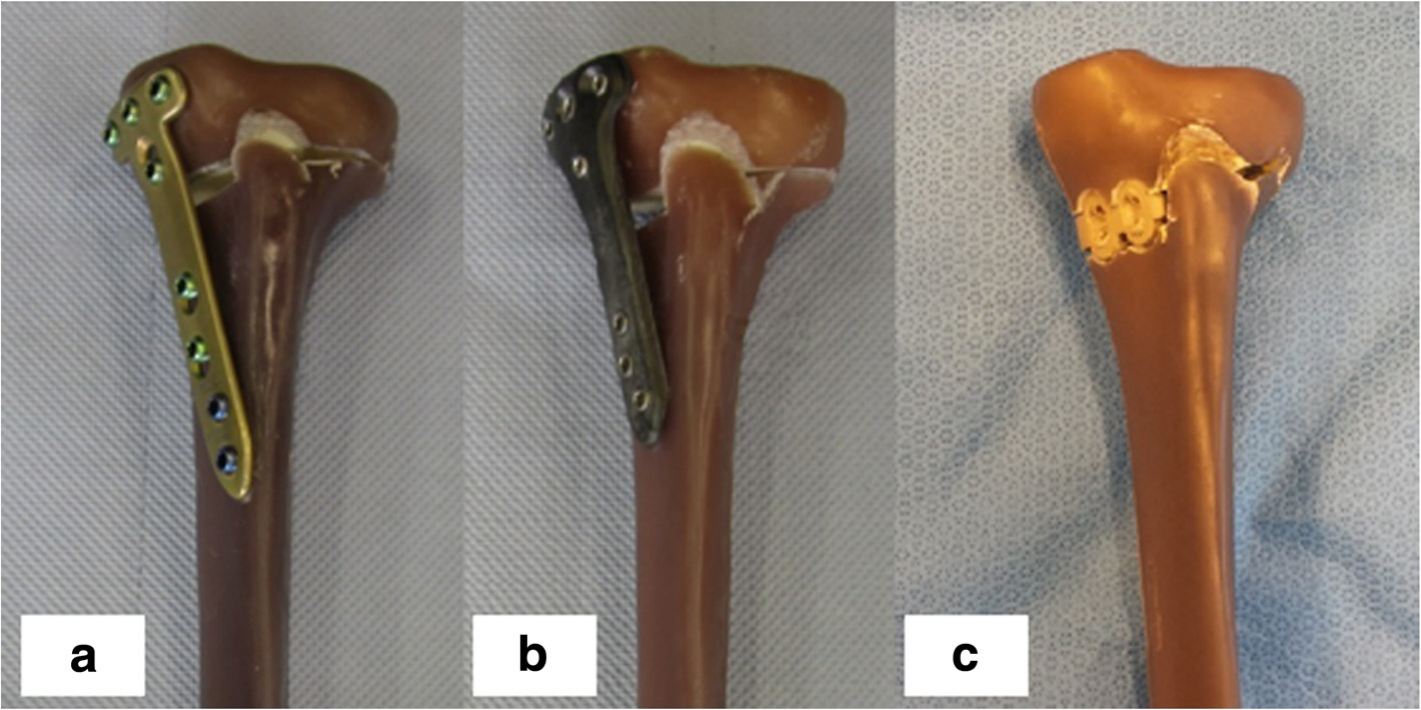
Fixation devices compared during the last tests performed. (**a**) TomoFix std plate, (**b**) PEEKPower HTO plate and (**c**) iBalance HTO implant. All this implants are indicated with no weight restriction

In our previously performed comparative biomechanical study (Maas et al. 2013) comparing the TomoFix sm plate and the Contour Lock plate (Arthrex, Munich, Germany) (Fig. 1) Static and fatigue strength for both plates were investigated. The same materials and methods were used in this study to perform static and cyclic tests on bone-implant constructs with the TomoFix std, the PEEK Power and the iBalance fixators (Fig. 2). The results obtained were then compared to those of our previous study, thus allowing a comparison between (1) the TomoFix sm plate, (2) the Contour Lock plate, (3) the iBalance implant, (4) the PEEK Power plate and (5) the TomoFix std plate. The hypothesis of the present study was that implants well attached with a wide proximal end to the tibial head provide better stability to the bone-implant construct.

Except for the iBalance system thermoplastic (PEEK), screws were used whereas all other fixation plates used metal screws (titanium alloy or stainless steel).

## Methods

Sixteen large-size fourth-generation composite analogue tibia bone models (Sawbones, Pacific Research Laboratories, Inc., Vashon Island, Washington, USA) were used in this study since these composite bones have mechanical properties similar to human bones (Chong et al. 2007; Gardner et al. 2010; Heiner 2008). Using artificial composite bones has the advantage of minimizing biomechanical variability between the specimens, which can arise when using cadaveric bones.

### Specimen preparation

**Fig. 3:**
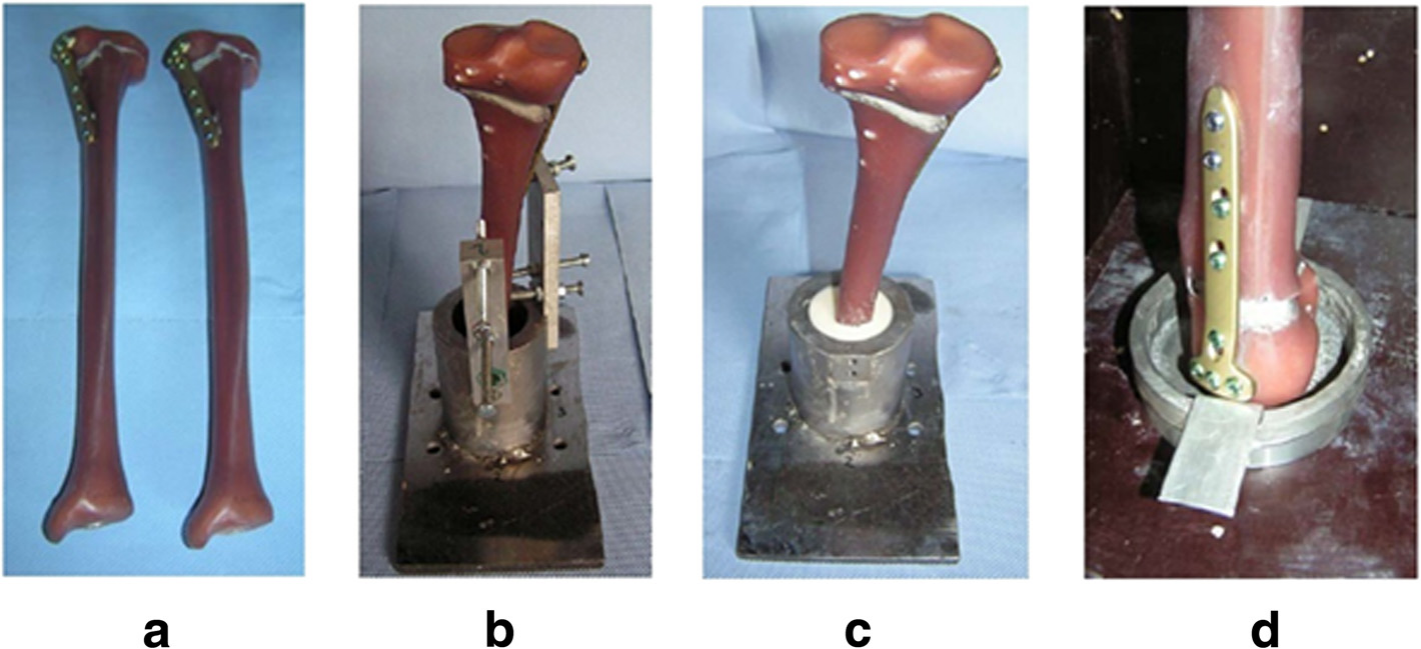
Different steps of a specimen preparation (Maas et al. 2013)**.** (**a**) The composite bones after the osteotomy; (**b**) Fixation in the cylindrical mold; (**c**) Specimen after casting with polyurethane resin FC 52; (d) Preparation of tibial head with pre-insertion of sensor attachments

Each specimen tested was prepared in the same standardized manner as described in the following four steps (Maas et al. 2013). (a) Open-wedge high tibial osteotomies were performed on each of the sixteen composite bones in the same way by an experienced surgeon, according to standard techniques of each implant (Fowler et al. 2000; Lobenhoffer and Agneskirchner 2003). (b) The composite tibias were then cut. Only the proximal part of 300 mm length was fixed in a cylindrical mold. All specimens were identically positioned with the help of a template and a centrical pinion at the bottom of the mold form. The inclination angle in the frontal and sagittal direction was such chosen that the tibial plateau was horizontal in both directions. The repeatability of the described positioning system was checked with different specimens and found to be less than 1 mm in all three dimensions. (c) The cylindrical pot was filled with a two-component polyurethane casting resin (FC 52) formed by reacting an isocyanate with a polyol in a 1:1 ratio (Huntsman Advanced Materials GmbH, Basel, Switzerland). (d) After the hardening of the resin, the specimens were turned 180° and the tibial heads with the osteotomy plates were positioned in shallow cylindrical molds. Two small thin-metal plates were added in the molds in order to attach the displacement sensors later on (Fig. 3).

### Mechanical testing system

**Fig. 4:**
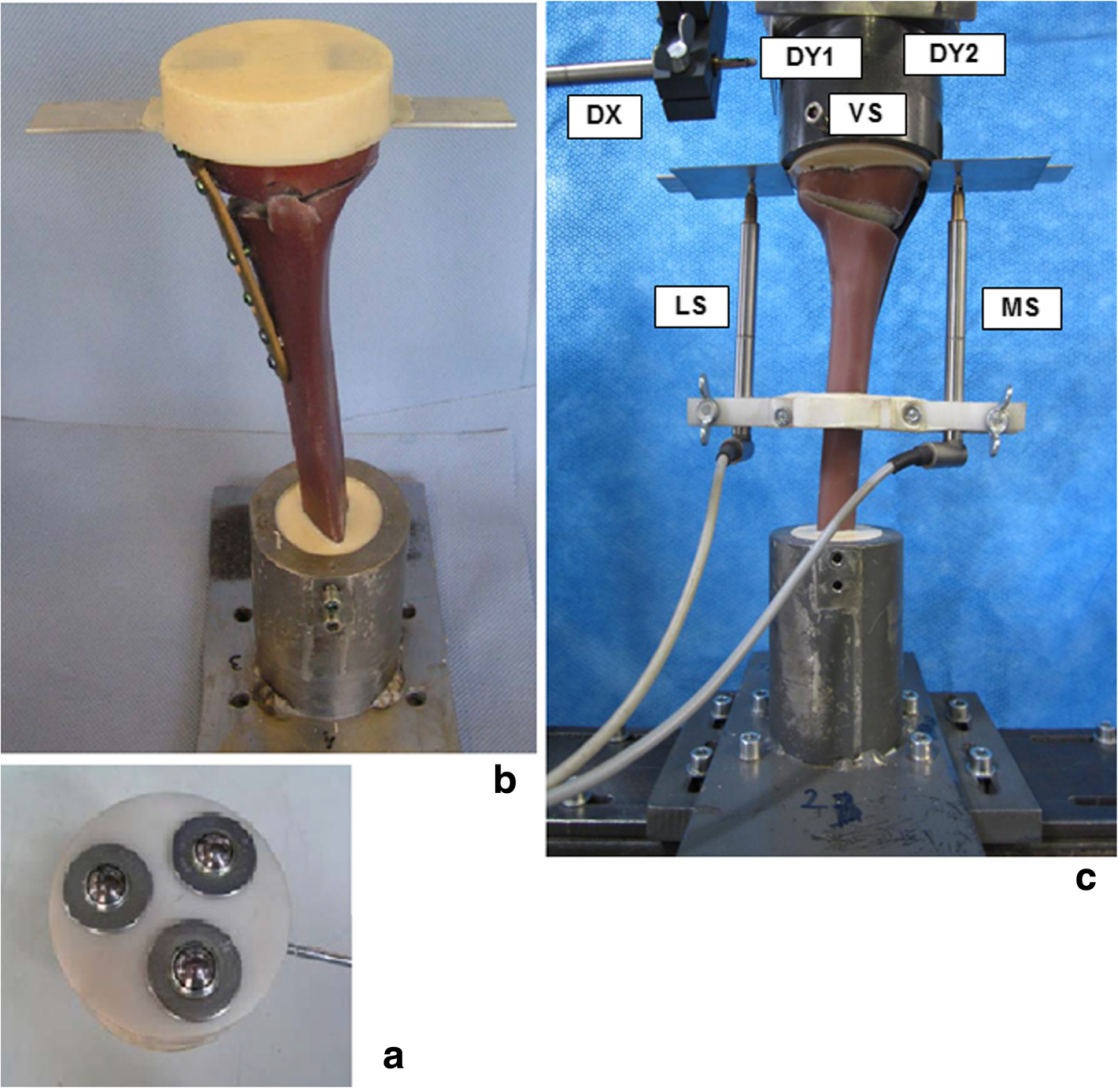
Materials and test setting-up. (**a**) Low friction sliding support to apply purely vertical forces. (**b**) Specimen before mounting to hydraulic press. (**c**) Specimen under test: the lateral and the medial sensor (LS and MS) registered the relative lateral and medial vertical displacements from the tibial head, while VS measured its vertical displacement. The DX, DY1 and DY2 sensors registered the horizontal displacements of the tibial head; along the transverse axis for the first and the sagittal axis for the latters

Purely vertical loading was applied to the tibia head through a freely movable support (Fig. 4-a), allowing only horizontal motion in the transversal plane, using three freely rolling metal balls. Hence a rotational movement or tilting of the tibia head was not possible. The tibial plateau always remained horizontal in both directions though no mechanical guiding rail was used. The deformations of the specimens were captured using six displacement sensors. A medial sensor MS measured the vertical displacement in the frontal plane on the medial side of the tibial head and the second sensor LS at the lateral side. The distal ends of the tibias were fixed to the vertical moving piston of the hydraulic testing machine with constrained motions in the transversal plane. Three displacement sensors DX, DY1 and DY2 were attached to the easily sliding support in order to measure the horizontal displacements of the tibial head in two horizontal perpendicular directions X (medial-lateral) and Y (proximal-distal) in the transverse plane. The sensors DY1 and DY2 were positioned in a way to detect rotation of the tibial head in the transversal plane. A sixth displacement sensor VS embedded in the testing hydraulic machine (INSTRON, Darmstadt, Germany) measured the vertical displacement of its piston (Fig. 4-c).

### Testing procedure

**Fig. 5:**
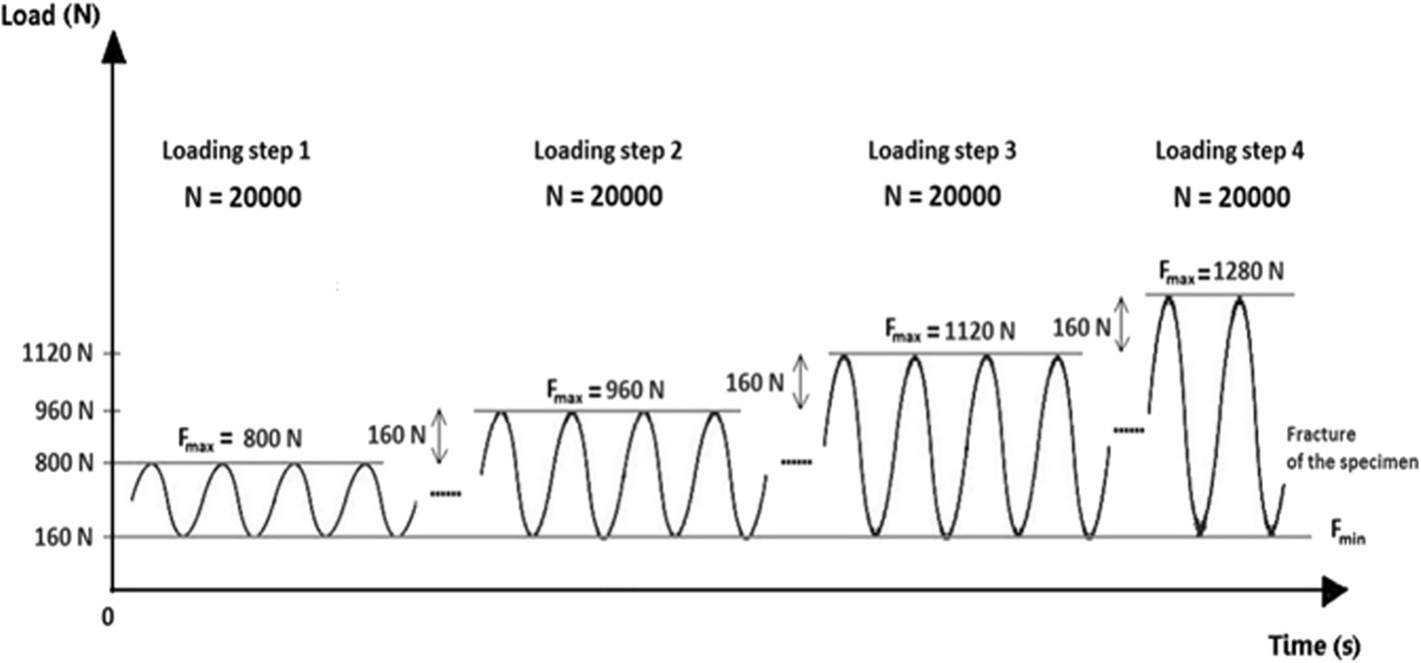
Scheme of the applied vertical sinusoidal force loading (load-controlled) (Maas et al. 2013). After N = 20000 cycles the upper force was increased stepwise by 160 N until failure. The loading frequency was constant and set to 5 Hz

The bone-implant-constructs were subjected to static and cyclic tests. The static tests consisted of applying quasi-static compression displacement-controlled single loading to failure at a speed of 0.1 mm/s. The dynamic tests consisted of load-controlled cyclic fatigue testing, with stepwise compression sinusoidal (frequency = 5Hz) loading where the force amplitude of each step was kept constant with feed-back control of the force signal within the hydraulic machine. The lower compressive force limit of each load step was kept constant at 160 N. Starting with 800 N for the first step, the upper compressive force limit was increased stepwise by 160 N after N = 20000 cycles if no failure occurred (Fig. 5). The failure criteria considered in this study are summarized in Table 1. The present testing procedure is similar to the standardized testing protocol for hip joints (ISO 7206-4, 1989; ISO 7206-6, 1992; ISO 7206-8, 1995) and was also used in a study by Agneskirchner et al. (Agneskirchner et al., 2006).

**Table 1:** The failure types used and their defining criteria

Failure type	Criteria
1	Medial or lateral displacements of the tibial head in relation to the tibial shaft greater than 2 mm. This criterion can only be checked in the unloaded condition.
2	Visible collapse of the lateral cortex. Small hairline cracks are not considered as failure.
3	Maximal displacement range greater than 0.5 mm within one hysteresis loop in the case of cyclic testing only.
4	Cracks of the screws greater than 1 mm

The sixteen specimens were regrouped as follows: group 1 (n = 5, TomoFix standard plates (TomoFix std)), group 2 (n = 5, PEEKPower plates) and group 3 (n = 6, iBalance implants). The groups were further subdivided depending on the performed test (Table 2). Group 4 (n = 5, TomoFix small stature plates (TomoFix sm)) and group 5 (n = 5, ContourLock plates) were composed from the specimens used in our previous study (Maas et al. 2013). It is important to recall here that the specimens used in the previous study were prepared in the same standardized manner as indicated in the previous paragraph.

**Table 2:** Specimen grouping and assignment, depending on the implants used and the tests performed

Test	Group 1; n = 5 Specimens	Group 2; n = 5 Specimens	Group 3; n = 6 Specimens	Group 4; n = 5 Specimens	Group 5; n = 5 Specimens
Static: Single loading to failure test	TomoFix 1	PEEKPower 1	iBalance 1	TomoFix sm 1	ContourLock 1
	TomoFix 2	PEEKPower 2	iBalance 2	TomoFix sm 2	ContourLock 2
Dynamic: cyclic fatigue failure test	TomoFix 3	PEEKPower 3	iBalance 3	TomoFix sm 3	ContourLock 3
	TomoFix 4	PEEKPower 4	iBalance 4	TomoFix sm 4	ContourLock 4
	TomoFix 5	PEEKPower 5	iBalance 5	TomoFix sm 5	ContourLock 5
			iBalance 6		

### Type and definition of failure

Defining the failure criteria is decisive since the specimens were never completely damaged. The failure criteria that were used in this biomechanical study are summarized in Table 1 and were already used by Pape et al. (Pape et al., 2010). With type 3 failure the wobble degree or the stability of the sample during the cyclic testing was quantified. This was checked by plotting the force versus the displacement; for nonlinear cases this plot is ideally an elliptical curve with a slope proportional to the stiffness and an enclosed area proportional to the damping of the structure tested. If the specimen becomes unstable and starts to wobble, e. g. after a failure, the width of this curve representing the maximal displacement range increases.

### Mechanical stiffness

**Fig. 6:**
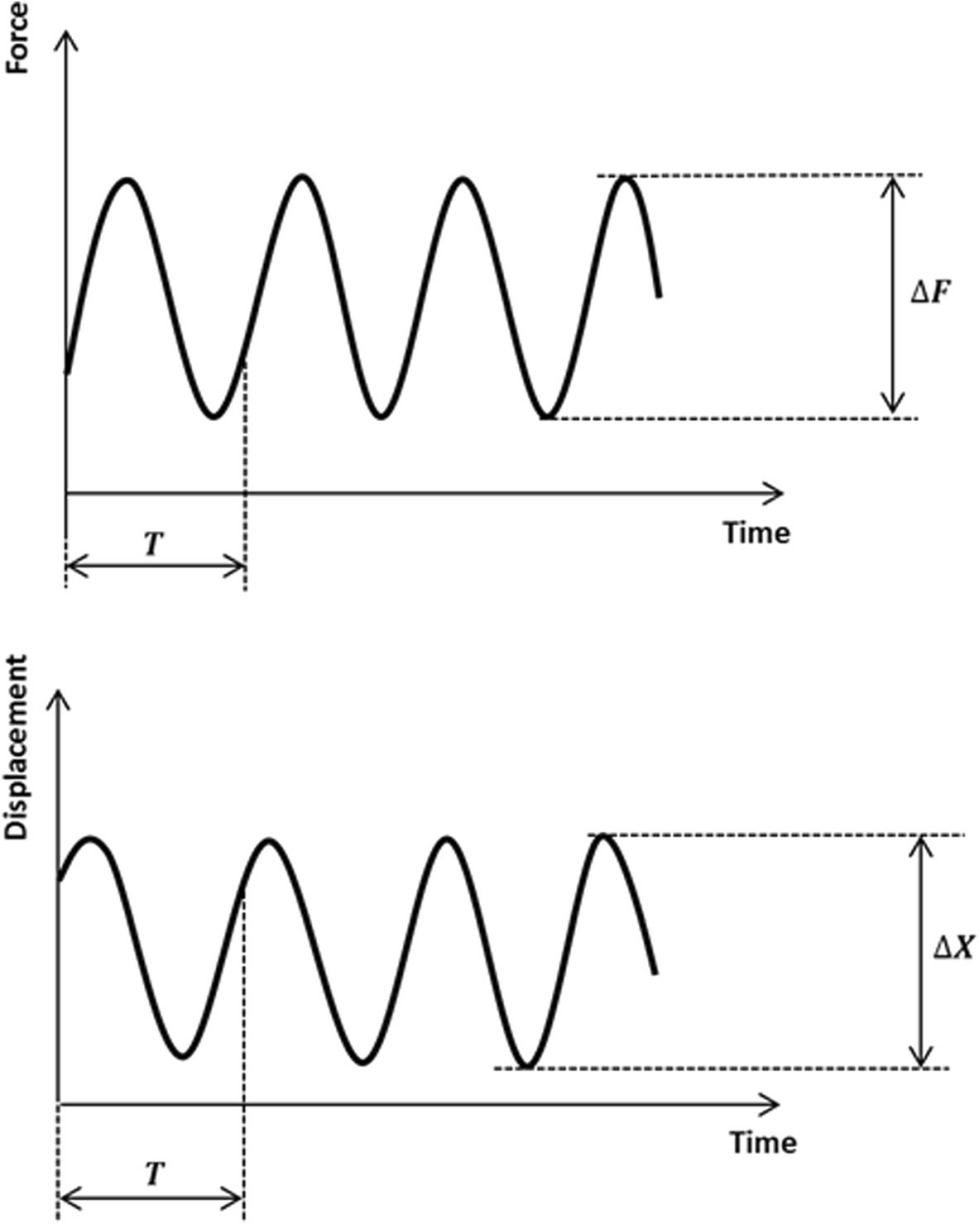
Definition of ΔF and ΔX to calculate dynamic stiffness in the cyclic fatigue to failure tests (Maas et al. 2013). ΔF and ΔX are the peak-to-peak force and the corresponding peak-to-peak displacement in the same period T. This allows to determine the time history of the specimen stiffness during loading

Changes in stiffness are frequently used as an additional damage indicator in fatigue tests of complex structures. Therefore the evolution of the dynamic stiffness of the specimens subjected to the cyclic testing was determined as the ratio of peak-to-peak force ∆F = F_max_-F_min_ to the peak-to-peak displacement ∆X measured in the same period T (Fig. 6):

$$ k=\frac{F_{\max }-{F}_{\min }}{\varDelta \mathrm{X}}. $$

Lateral and vertical stiffness were defined by considering the lateral and the vertical displacements registered by the lateral (LS) and the vertical (VS) sensors respectively. Medial stiffness depends on the implants’ stiffness and is thus not considered.

For the static loading to failure test static stiffness was defined as the ratio of the measured force to the measured displacement at a given time.

### Permanent plastic deformation of the specimen

**Fig. 7:**
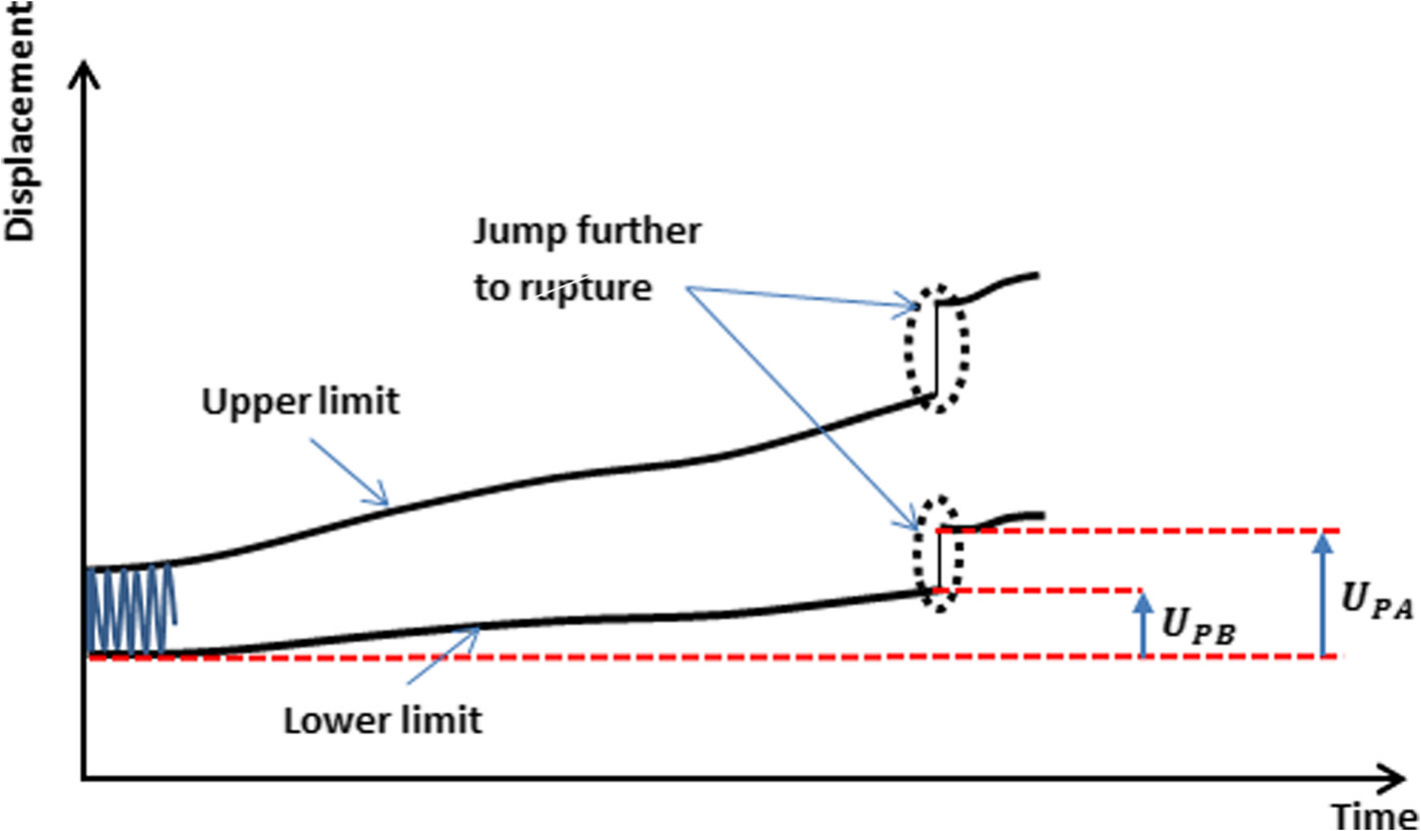
Definition of permanent plastic deformation before and after failure (Maas et al. 2013): creep deformations of the specimens were observed. This allowed defining permanent plastic deformations before (UPB) and after failure (UPA) as the irrecoverable displacement with respect to the initial instantaneous displacements

Any permanent plastic deflection and the correlated permanent plastic deflection angle would lead to an alteration of correction and had to be checked to quantify type 1 failure as the criterion can only be checked in the unloaded condition. Permanent plastic deformation (Fig. 7) was estimated here as the irrecoverable displacement with a minimal force of 160 N, considered as nearly zero force, at the start of the tests. Permanent plastic deformations could therefore be measured online during the cyclic tests at any time, for example before failure (U_PB_) and additionally after the gross failure, i.e. the collapse of the lateral cortex (U_PA_) in general.

The permanent plastic valgus malrotation of the tibial head, which corresponds to the permanent plastic deflection angle of the tibial plateau at a given time, was defined as the resulting permanent plastic displacements on the medial and the lateral sides in the specimens’ frontal plane.

**Fig. 8:**
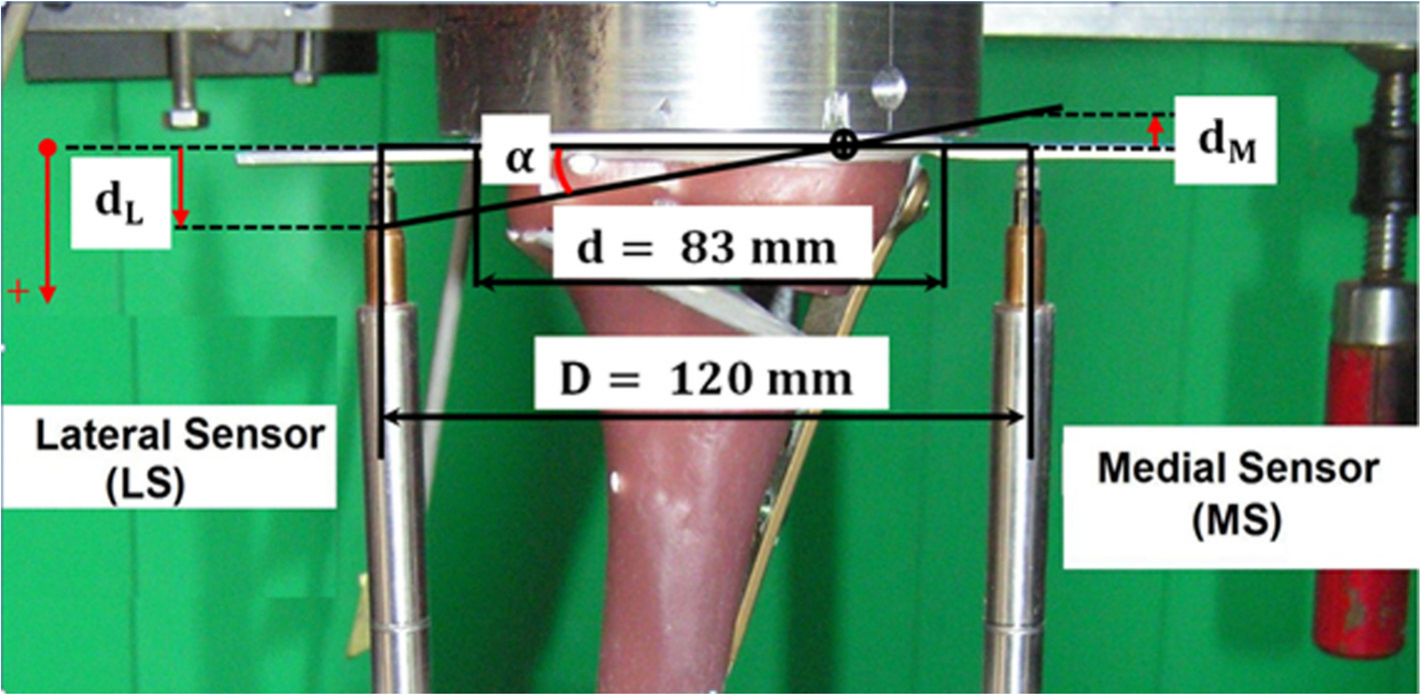
Definition of the deflection angle (Maas et al. 2013). In general the lateral displacement d_L_ was found to be positive with greater magnitude than the medial displacement d_M_ that was counted as negative. Hence the deflection angle *α* was defined by means of the difference (d_L_-d_M_)

As already indicated, the two sensors LS and MS registered the lateral displacement d_L_ and the medial displacement d_M_ of the tibial head respectively. Though the test force was strictly vertical, it was observed that the specimens deformed not purely vertically, which resulted in unequal displacement values for d_L_ and d_M_ (Fig. 8). The valgus malrotation of the tibia was then defined as the deflection angle (in radians) in the frontal plane and could be calculated at any time as

$$ \upalpha =\frac{\left|{d}_L-{d}_M\right|}{D}. $$

According to the definitions of permanent plastic deformations type 1 failure occurs when

$$ \frac{\mathrm{d}}{\mathrm{D}}\left|{\mathrm{d}}_{\mathrm{Lp}}-{\mathrm{d}}_{\mathrm{Mp}}\right|>2\ \mathrm{mm}; $$

that is if α_p_ > 0.024 rad or 1.4°, with the index “*p*” meaning permanent plastic.

The displacements in the direction of the loading (e.g. the vertical descending direction) were considered as to be positive and negative in the opposite direction.

## Results and discussion

### Results

#### Static loading to failure tests

**Fig. 9:**
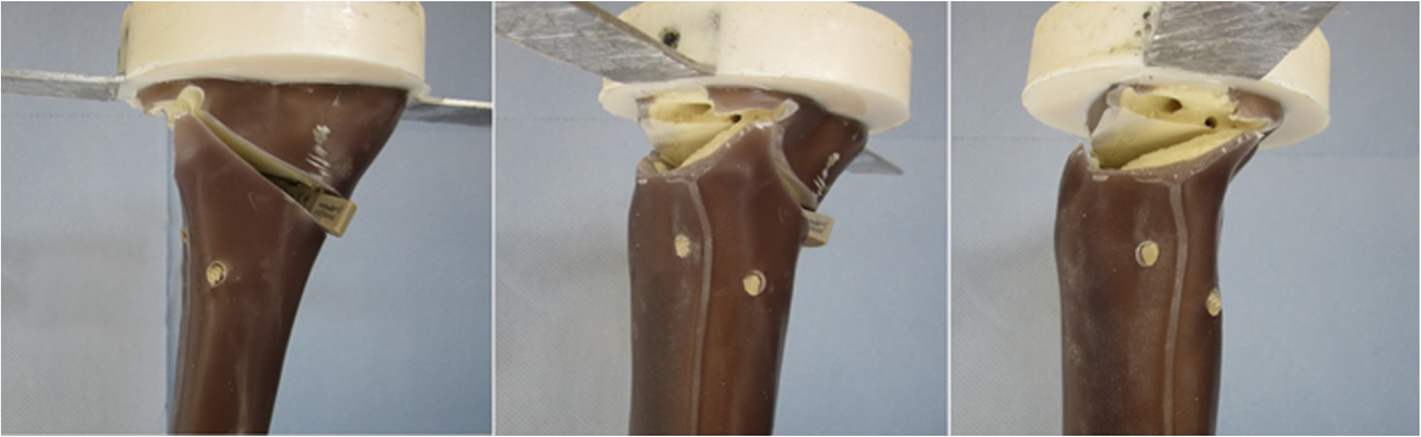
Fracture of the contralateral cortex (specimen with the iBalance implant). The opposite cortex appeared to be the weak point of the bone-implant constructs

**Fig. 10:**
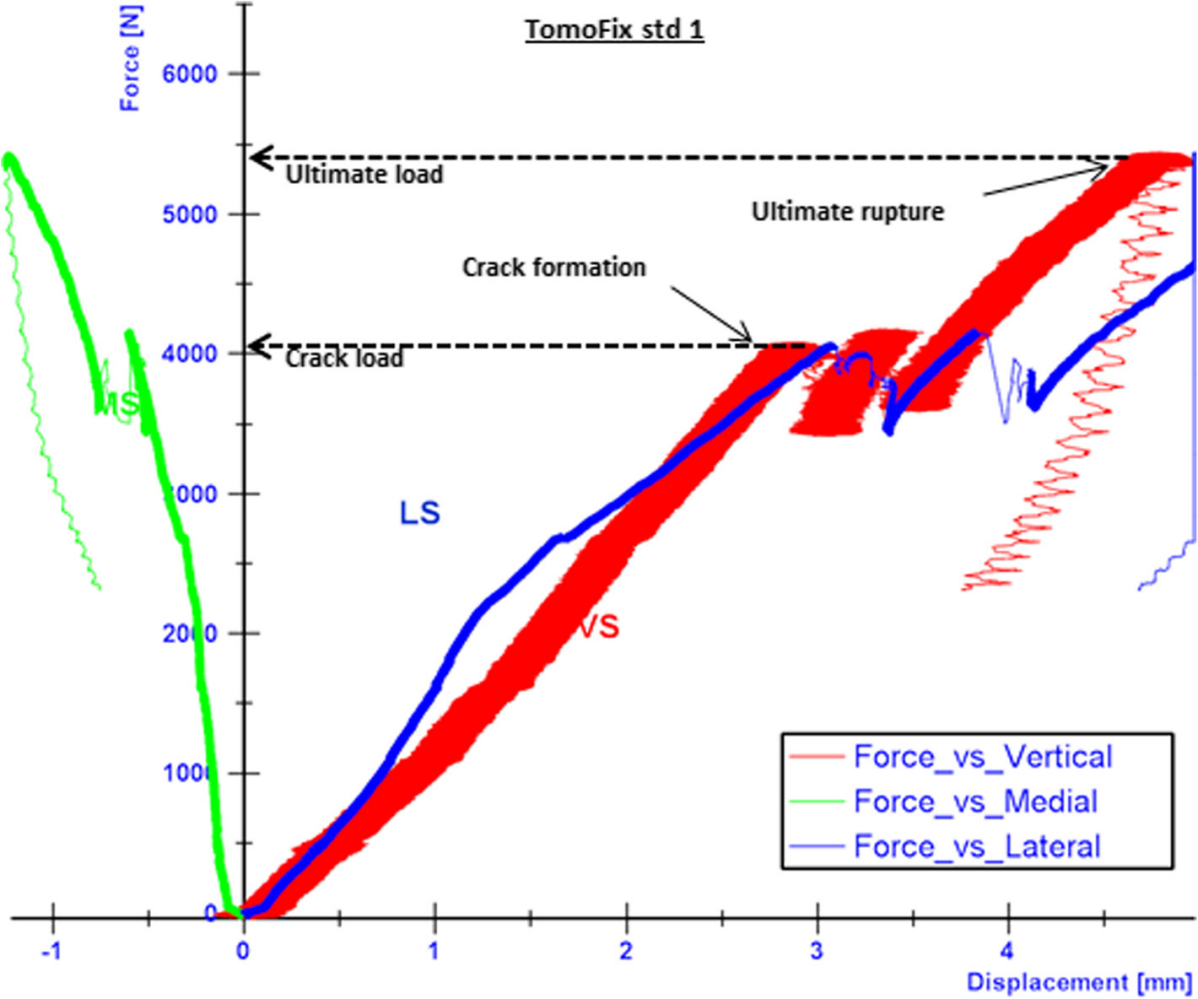
Curves of the force versus the displacements in cases of TomoFix std1. The final rupture of the specimen occurred quite promptly after the crack formations, which were characterized by a drop in the registered applied force. The crack and the ultimate loads were considered as the approximate loads at the moment the crack formations and collapse of the contralateral cortex occurred respectively

**Fig. 11:**
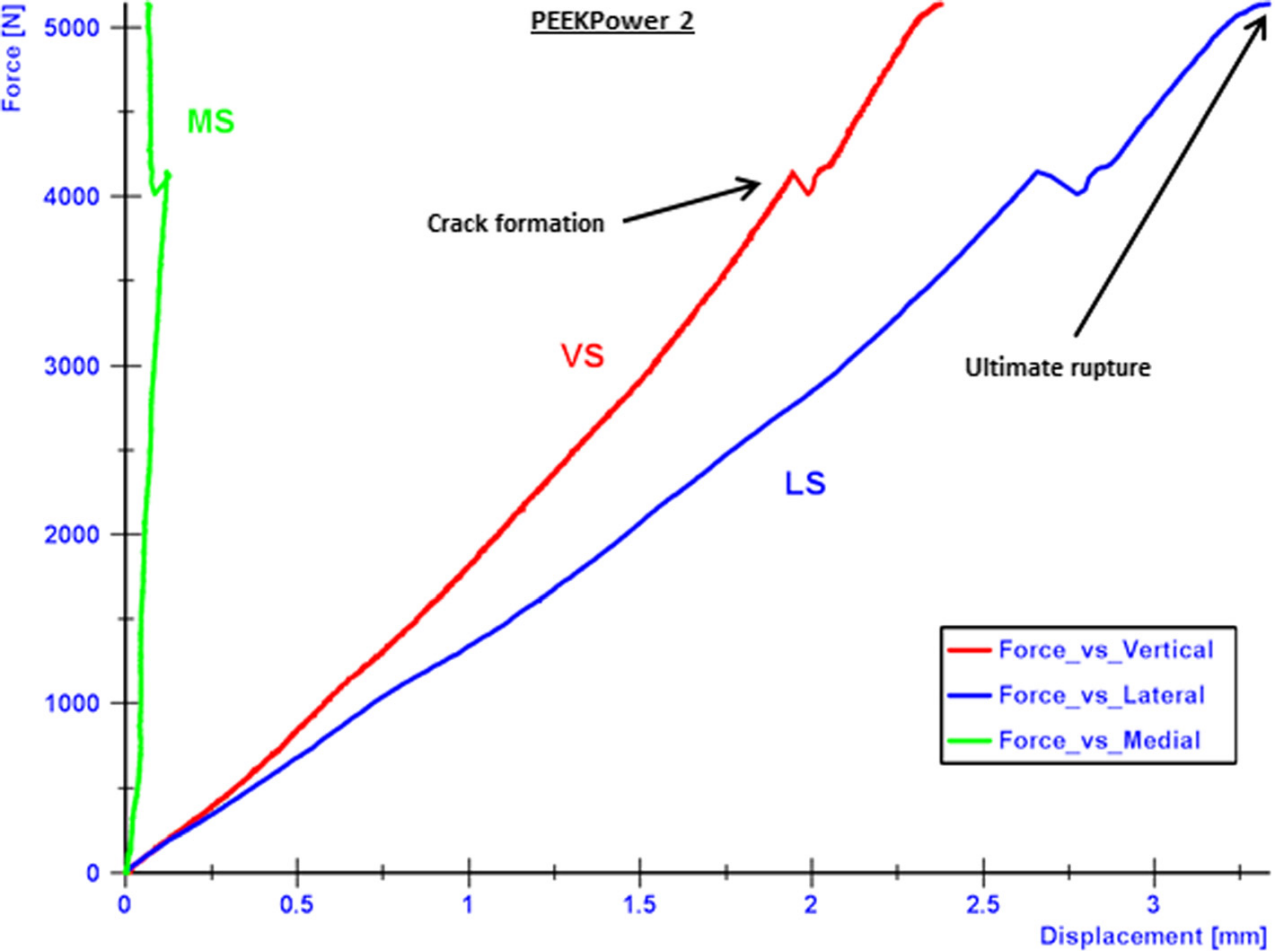
Curves of the force versus the displacements in cases of PEEKPower 2. The final rupture of the specimen occurred quite promptly after the crack formations. The medial displacement (MS) was positive contrary to e.g. the TomoFix std 1

All specimens failed due to a fracture of the contralateral cortical bone, which represents type 2 failure (Fig. 9). Before the final collapse there were crack formations observed in the cases of the TomoFix std 1 (Fig. 10) and PEEKPower 2 (Fig. 11). For the others specimens the abrupt fractures of the contralateral cortex were not preceded by observable crack formations.

While considering the vertical descending displacements as positive, the medial displacement (MS) was negative in the cases of the TomoFix std 1 (Fig. 10), TomoFix std 2, PeekPower 1 and iBalance 2. The lateral displacement (LS) was always positive and its absolute value remained greater than the medial displacement. The same applies when the latter was positive for PEEKPower 2 (Fig. 11) and iBalance 1, meaning that the tibial plateau of all specimens rotated during the static loading.

The crack and ultimate loads of the specimens were determined as indicated in Fig. 10 and are summarized with the corresponding medial and lateral displacement, valgus malrotation of the tibial head and static lateral stiffness in Table 3. The values of the specimens of groups 4 and 5 (TomoFix sm and ContourLock) are from our previous study and presented here for sake of comparison.

**Table 3:** Static tests summary: displacements, valgus malrotation of the tibial head and their corresponding cracks and ultimate loads, including mean values and standard deviations (SD)

Specimen	Crack / ultimate load [kN]	Medial displ. at crack / ultimate load [mm]	Lateral displ. at crack / ultimate load [mm]	Valgus malrotation of the tibial head at crack / ultimate load (°)	Lateral stiffness at crack / ultimate load [kN/mm]	Failure types
TomoFix std 1	4.1 / 5.4	0.6 / 1.2	3.1 / 5.0	1.8 / 2.9	1.3 / 1.1	1 and 2
TomoFix std 2	5.1 / 5.2	1.0 / 1.1	4.2 / 4.4	2.5 / 2.6	1.2 / 1.2	1 and 2
Mean:	4.6 / 5.3	0.8 / 1.2	3.7 / 4.7	2.1 / 2.8	1.3 / 1.1	
SD ±:	0.7 / 0.1	0.3 / 0.1	0.8 / 0.4	0.5 / 0.2	0.1 / 0.1	
PEEKPower 1	-/ 3.7	-/ 0.5	-/ 2.9	-/ 1.6	-/ 1.3	1 and 2
PEEKPower 2	4.2 / 5.1	0.1 / 0.1	2.7 / 3.3	1.3 / 1.5	1.6 / 1.5	1 and 2
Mean:	-/ 4.4	-/ 0.3	-/ 3.1	-/ 1.6	-/ 1.4	
SD ±:	-/ 0.1	-/ 0.3	-/ 0.3	-/ 0.1	-/ 0.1	
iBalance 1	-/ 5.7	-/ 0.3	-/ 1.6	-/ 0.6	-/ 3.6	2
iBalance 2	-/ 5.4	-/ 0.3	-/ 2.1	-/ 1.1	-/ 2.6	2
Mean:	-/ 5.5	-/ 0.3	-/ 1.9	-/ 0.9	-/ 3.1	
SD ±:	-/ 0.2	-/ 0	-/ 0.4	-/ 0.4	-/ 0.7	
TomoFix sm 1	3.1 / 3.2	0.6 / 0.9	1.3 / 1.8	0.9 / 1.3	2.4 / 1.8	2
TomoFix sm 2	3.2 / 3.6	0.4 / 0.6	1.6 / 2.3	0.9 / 1.4	2.0 / 1.6	2
Mean:	3.2 / 3.4	0.5 / 0.8	1.5 / 2.1	0.9 / 1.4	2.2 / 1.7	
SD ±:	0.1 / 0.3	0.1 / 0.2	0.2 / 0.4	0 / 0.1	0.3 / 0.1	
ContourLock 1	2.4 / 3.2	0.6 / 0.5	2.5 / 3.9	1.5 / 2.1	1.0 / 0.8	1 and 2
ContourLock 2	-/ 3.9	-/ 0.5	-/ 4.2	-/ 2.2	-/ 0.9	1 and 2
Mean:	-/ 3.6	-/ 0.5	-/ 4.1	-/ 2.2	-/ 0.9	
SD ±:	-/ 0.5	-/ 0	-/ 0.2	-/ 0.1	-/ 0.1	

There were no cracks observed prior to the ultimate failure in the case of PEEKPower 1, iBalance 1 & 2 (all values rounded to the 1st decimal)

The PEEKPower 2, ContourLock 1, TomoFix std 1 & 2 and ContourLock 2 specimens showed the largest lateral displacements at collapse time. The average displacement on the medial compared to the lateral side was always smaller for all implant types. The determined valgus malrotation of the tibial head was greater or equal to the fixed limit of 1.4° of the permanent deflection angle for all implants, except for the iBalance specimen which showed a mean value of 0.9° (Table 3). The iBalance specimens were the stiffest bone-implant constructs for the performed static loading to failure tests with an average lateral stiffness of 3.1 kN/mm at ultimate load.

#### Fatigue loading to failure tests

**Fig. 12:**
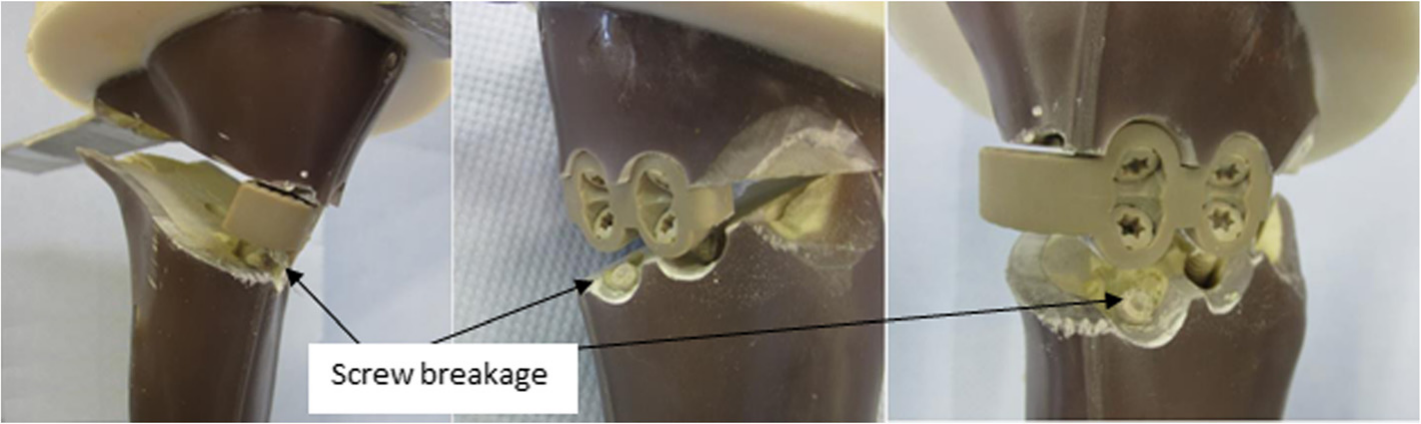
Damage of the iBalance 3 specimen. The iBalance implant damaged once during the cyclic test. Ruptures of the screws were observed. There was only one type 4 failure observed

As for the static tests all the specimen failed in the fatigue tests due to the fracturing of the contralateral cortical bone. Damage of the implant occurred only in the case of the iBalance 3, for which a rupture of the screw and fracture of the medial cortex as indicated in Fig. 12 was observed.

**Fig. 13:**
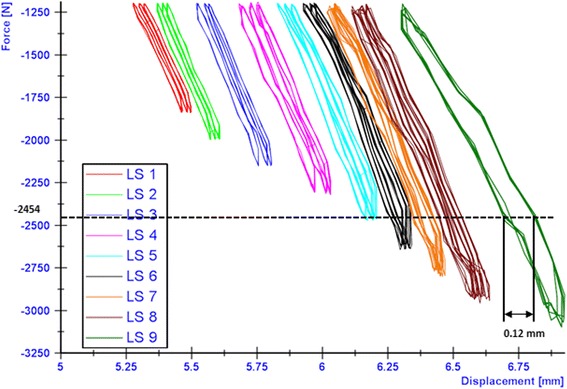
Hysteresis loops: lateral displacement (iBalance 5 specimen). The higher the loads, the more the maximal displacement range within the loops grew but was never bigger than 0.5 mm

**Fig. 14:**
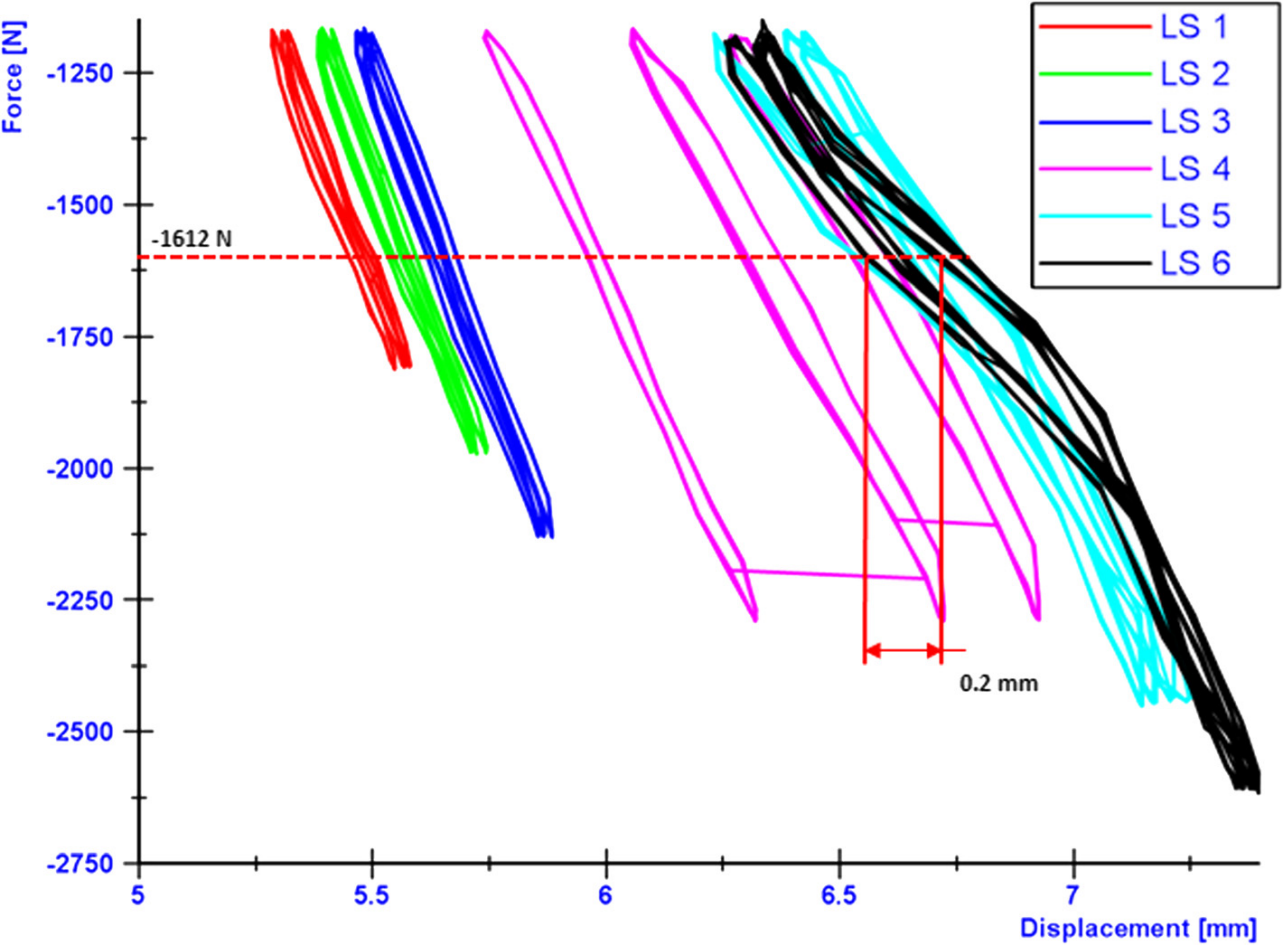
Hysteresis loops: lateral displacement (PEEKPower 5 specimen). The higher the loads, the more the maximal displacement range within the loops grew but was never bigger than 0.5 mm

**Fig. 15:**
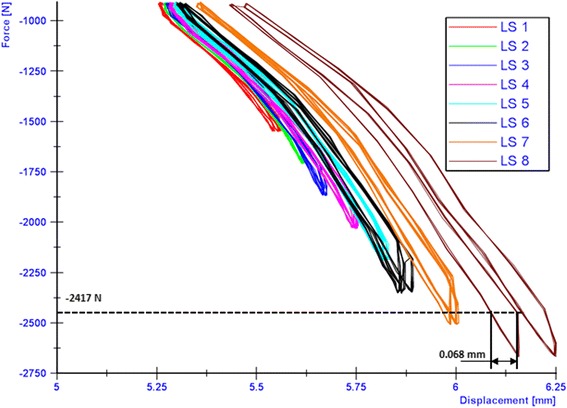
Hysteresis loops: lateral displacement (TomoFix 5 specimen). The higher the loads, the more the maximal displacement range within the loops grew but was never bigger than 0.5 mm

The breakage of the screw occurred at the transition zone of elasticity modulus difference between the stiffer cortical bone and the PEEK screw. The applied loads versus the resulting lateral displacements were plotted in order to check type 3 failures (Table 1). The maximal displacement ranges within the hysteresis loops were determined as showed by the examples in Figs. 13, 14 and 15 in cases of the iBalance 5, PEEKPower 5 and TomoFix std 5 respectively. The values obtained were always below the predefined limit of 0.5 mm. Hence there was no type 3 failure observed for the specimens of group 1, 2 and 3. The lateral displacement (LS) was always used since it was higher than the medial displacement (MS) and the rupture occurred always on that side.

**Fig. 16:**
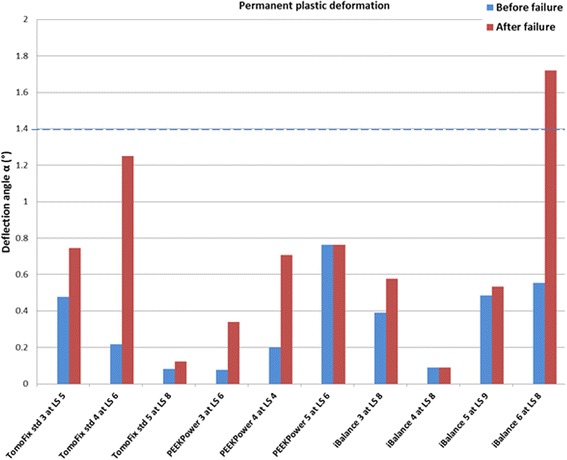
Deflection angle or valgus malrotation of the tibial head before and after failure for groups 1, 2 and 3. A type 1 failure was only observed in the case of the iBalance 6 specimen after the collapse of the opposite cortex

**Fig. 17:**
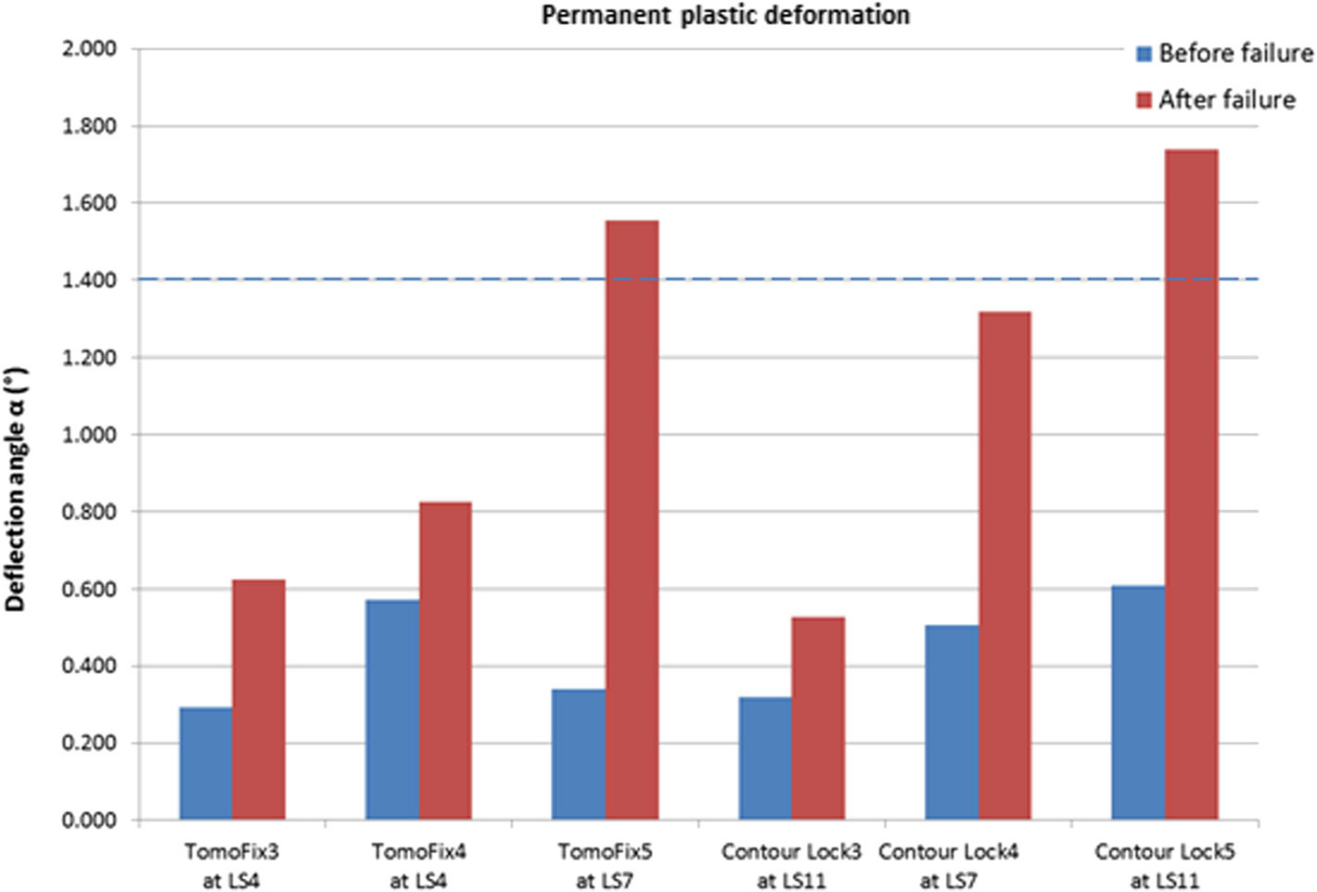
Deflection angle or valgus malrotation of the tibial head before and after failure for groups 4 and 5 (the graph is retrieved from our previous study and showed here for sake of comparison). The TomoFix specimens here are the TomoFix small stature of group 4 in the present study

Permanent plastic valgus malrotations just before and after the collapse of the lateral cortex had been determined for the specimens of the first three groups (TomoFix std, PEEKPower and iBalance) and are represented in Fig. 16. The load history according to Fig. 5 is indicated with the Load Step number (LSn) at which the failure occurred. As stated above, a type 1 failure occurred when the permanent plastic valgus malrotation was greater than 1.4°. Only the iBalance 6 exceeded this limit, but only after the failure of the specimen. The graph in Fig. 17 retrieved from our previous study and presented for sake of comparison shows the permanent plastic valgus malrotations for the TomoFix sm and ContourLock specimens.

Tables 4 and 5 summarize the results of the cyclic fatigue to failure tests by listing the maximal compressive force, lateral and the vertical stiffness of the specimens at the beginning of the first load step, the number of cycles performed prior to failure and the types of failure. The data displayed in Table 5 are from our previous study. Table 6 shows mean values, including the standard deviations, per group of the characteristic values given in Tables 4 and 5 of the individual specimens.

**Table 4:** Summary of fatigue failure tests (groups 1, 2 & 3): max. load, vertical & lateral stiffness, number of cycles (all values prior to failure) and failure types

Specimen	Maximal load [N]	Vertical stiffness K_V_ [N/mm]	Lateral stiffness K_L_ [N/mm]	Number of cycles	Failure types
TomoFix std 3	1280	1350	2000	>60 000	2
TomoFix std 4	1440	2000	2500	>80 000	2
TomoFix std 5	1760	2500	2200	>120 000	2
PEEKPower 3	1440	2000	2500	>80 000	2
PEEKPower 4	1280	1950	2140	>60 000	2
PEEKPower 5	1440	2785	2250	>80 000	2
iBalance 3	1760	4000	3600	>120 000	2,4
iBalance 4	1760	3000	3400	>120 000	2
iBalance 5	1920	3000	2952	>140 000	2
iBalance 6	1760	3500	2500	>120 000	1,2

**Table 5:** Summary of fatigue to failure tests (groups 4 & 5): max. load, vertical & lateral stiffness, number of cycles (all values prior to failure) and failure types

Specimen	Maximal load [N]	Vertical stiffness K_V_ [N/mm]	Lateral stiffness K_L_ [N/mm]	Number of cycles	Failure types
TomoFix sm 3	1280	2200	2000	>60 000	2,3
TomoFix sm 4	1280	1750	1500	>60 000	2,3
TomoFix sm 5	1760	2000	2300	>120 000	1,2
ContourLock 3	2400	2100	4400	>200 000	2
ContourLock 4	1760	2300	2400	>120 000	2
ContourLock 5	2400	2700	2600	>200 000	1,2,3

From our previous study and reported here for purposes of comparison

**Table 6:** Average mean values, including the standard deviations (SD) per group of the cyclic fatigue to failure tests (all decimal values rounded to the 1st decimal)

Groups	Maximal load [kN]		Vertical stiffness K_V_ [N/mm]		Lateral stiffness K_L_ [N/mm]		Number of cycles prior to failure	
	Mean	SD ±	Mean	SD ±	Mean	SD ±	Mean	SD ±
TomoFix std	1.5	0.2	1950	577	2233	252	>86 000	30 550
PEEKPower	1.4	0.1	2245	468	2297	184	>73 000	11 500
iBalance	1.8	0.1	3375	479	3113	490	>125 000	10 000
TomoFix sm	1.4	0.3	1983	184	1933	330	>80 000	28 300
ContourLock	2.2	0.4	2367	250	3133	900	>173 000	37 700

**Fig. 18:**
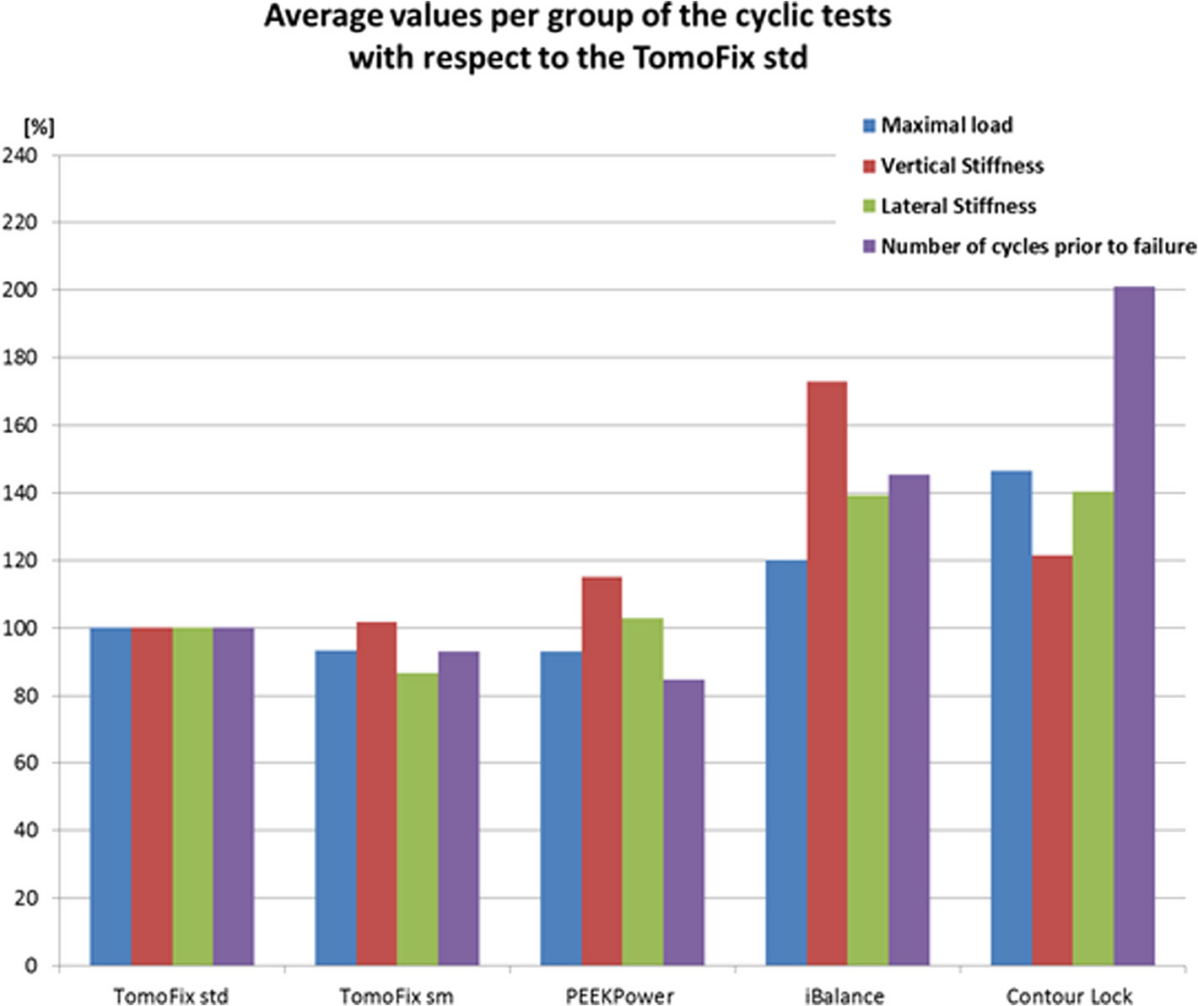
Average relative strength values of Table 6. The TomoFix std group was taken as reference

Based on Table 6 and taking the TomoFix std group as reference, average relative values per groups of the cyclic tests are shown in Fig. 18. Regarding the parameters investigated for cyclic tests, the iBalance group showed the highest values compared to groups 1 and 2. The PEEKPower group showed higher stiffness compared to the TomoFix plates. The vertical stiffness of the iBalance group was on average around 1.7 higher than the one of the TomoFix std group.

### Discussion

In this study three implants were investigated and compared to our previous study using the same experimental setup and protocol, thus comparing the static and fatigue fixation stability provided by the following five different medial open-wedge HTO plates: the TomoFix std plate, the PEEK Power plate, the iBalance implant, the Contour Lock HTO plate and the TomoFix sm plate. The key findings of the present study were the following: (1) the stiffest bone-implant construct was found to be the iBalance implant followed by the Contour Lock plate. (2) The Contour Lock plate provided highest fatigue strength under cyclic loading conditions. (3) Static loading until failure tests revealed superior strength for the iBalance implant followed by the TomoFix std, the PEEK Power plate, the Contour Lock and the TomoFix sm plates. (4) All implants withstood the maximal physiological vertical tibiofemoral contact force while slow walking. This force corresponds to about three times the body weight (Heinlein et al. 2009; Taylor et al. 2004), e.g. 2400 N for a patient weighing 80 kg.

The principal failure mode of all bone-implant-constructs tested was the collapse of the opposite cortex, which generally occurred quickly after the initial opposite cortex crack. This behavior was observed regardless whether a static or cyclic failure test was applied. This finding correlates with previous studies with the lateral cortex being a medial HTO’s weakest point (Spahn and Wittig 2002; Stoffel et al. 2004; Zhim et al. 2005; Agneskirchner et al. 2006; Maas et al. 2013; Watanabe et al. 2014). The displacements of the lateral side of the osteotomy were more pronounced than the medial displacement, which explains the valgus rotation in the frontal plane of the tibial head during the static and the cyclic loading tests.

The mean ultimate forces were 5.5 kN, 5.3 kN, 4.4 kN, 3.6 kN and 3.4 kN for the iBalance, the TomoFix std, the PEEK Power, the Contour Lock and the TomoFix sm group respectively. Hence, the iBalance and the TomoFix std are superior regarding static performance. The iBalance implant showed however the smallest mean lateral displacement (1.9 mm) at the time of opposite cortex fracture indicating a certain superiority compared to the TomoFix std plates, which failed at the largest mean lateral displacement of 4.7 mm and the largest valgus malrotation of the tibial head of 2.8° thus exceeding the limit of 1.4°, which corresponds to the occurrence of type 1 failure.

Static loading simulates only the standing of a patient. After medial HTO however, patients are also supposed to perform calm activities. The repetitive loadings of the knee are better simulated by fatigue tests. The maximal loads at failure observed during the fatigue tests were all smaller than the threshold value of 2.4 kN (Table 6). Full loading of the knee directly after an osteotomy should therefore be avoided. When considering these maximum loads at failure and the number of cycles carried out, the ContourLock plate performed best with 2.2 kN and 173000 cycles and the PEEKPower plate worst with 1.4 kN and 73000 cycles. Based on these two parameters a ranking for the dynamic analysis would place the iBalance in the second position after the ContourLock, followed by the TomoFix std and the TomoFix sm.

The maintenance of the primary correction is decisive for a positive outcome of the osteotomy. When considering the deformation mode of the specimens during the cyclic loading, a permanent plastic valgus malrotation of the tibial head occurred which caused a valgus deformation of the knee, consequently altering the localization of the mechanical axis and the primary performed correction. The valgus malrotation of the tibial head can be considered as an overcorrection. There was no permanent plastic valgus malrotation of the tibial head observed before the collapse of the specimens, just for the iBalance 6 specimen after failure (Fig. 16). As shown in Fig. 17 retrieved from (Maas et al. 2013), permanent plastic valgus malrotation after failure was observed in the TomoFix sm (group 4) and ContourLock (group 5) groups. As a result we can assume that the TomoFix std and the PEEKPower plates conserve correction better. Therefore the number of load steps performed before failure needs also be taken into account. However this observation is only valid if there was no bone healing prior to the fatigue failure, which is not a realistic scenario.

As indicated in Figs. 13, 14 and 15 the hysteresis loops are growing with damage but do not reach the critical threshold of 0.5 mm (Table 1) for the specimens of the TomoFix std, PEEKPower and iBalance groups. Maas et al. (Maas et al. 2013) reported the occurrence of type 3 failure for the ContourLock group (group 5) and twice within the TomoFix sm (group 4). Thus they concluded that the ContourLock plate was superior to the TomoFix sm plate as far as this parameter is concerned. This result was correlated to the fact that the ContourLock plate is wider and that the fixation screws are more widely spaced than those of the TomoFix sm. In other words, the ContourLock plate is better anchored to the tibial head.

The high mechanical strength of the iBalance construct is most likely due to its higher stiffness compared to other specimens since it is screwed within the osteotomy gap. It thus fills a part of the gap and provides a closed-wedge design with a higher stiffness compared to all other bone-implant constructs (Table 6 and Fig. 18). However there was damage of the iBalance system’s screws observed at very high number of cycles and loading. This underlines the importance of a good positioning of the screws and their anchorage in the tibial head. Though the loading was only vertical the screws and fixation had undergone complex tridimensional loading which is surely even more pronounced in reality than it was in this simplified test. It should be repeated that the screws of the iBalance are thermoplastics (PEEK) and not metallic materials like for the other fixation systems tested.

Agneskirchner et al. (Agneskirchner et al. 2006) also performed static compression load to failure and cyclical load to failure tests using composite bones and compared the stability of four different implants: three spacer plates and a TomoFix plate. The spacer plates were shorter than the TomoFix plate. The loads were also applied vertically to the bone-implant-constructs as it was done in the present study. They reported longer resistance to failure of the specimens for the TomoFix during the static tests. Stoffel et al. (Stoffel et al. 2004) also performed axial compression load to failure tests to compare the TomoFix plate to the Puddu plate (rectangular short spacer plate) by using composite bones and also reported a better axial stability for the TomoFix. Watanabe et al. (Watanabe et al. 2014) also performed a comparative study of the TomoFix and the Puddu plates by using cadaveric bones and reported the highest failure load for the TomoFix. None of the previously cited studies included the iBalance and the PEEKPower implants, whereas we did not include the Puddu plate. The TomoFix plate fixator yielded the best fatigue strength during the cyclic load to failure tests performed by Agneskirchner et al. (Agneskirchner et al. 2006), who reported that a rigid long plate fixator with stable locking bolts provides the best results in open-wedge HTO. Maas et al. (Maas et al. 2013) reported higher fatigue strength for the ContourLock plates when compared to the TomoFix sm plates. This observation diverges somewhat with the findings of Agneskirchner et al. (Agneskirchner et al. 2006). With regard to the results of our study, it can be concluded that the ContourLock was also superior to the TomoFix std regarding the cyclic load to failure tests (Table 6 and Fig. 18). Furthermore the iBalance group showed superior stability than the TomoFix groups in the cyclic loading hence one may conclude that stability provided by a plate is not correlated only to its length, but also to the kind of fixation, which seems to be the governing feature since the plates already have sufficient stiffness and strength.

Clinical studies reported better clinical results in terms of implant-related complications, non-unions and stability (Saeed and Rae 2009; Valkering et al. 2009; Woon-Hwa et al. 2013; Cotic et al. 2014; Staubli et al. 2003) for the TomoFix plates. This high healing rate is supposedly due to the callus-massage effect of the implant, meaning no compression between plate and bone (Staubli et al. 2003), which promotes osteogenesis thanks to the elastic bone-implant construct (Staubli 2008; Staubli and Jacob 2010). This fact is in accord with the present results since the TomoFix plates did not show the highest stiffness in this study. Nevertheless the question of the correlation between the best osteotomy outcomes and the stiffness provided by the implants could be discussed.

The limited number of specimens per group should also be taken into account. The testing procedure differs from reality where muscle forces, bending moments and ligament act together in the knee biomechanics and hence alter the real charging of the plates. As already stated by (Brinkman et al. 2014), it is important to consider life-like test conditions while performing biomechanical testing of implants. The loads applied during the cyclic fatigue testing resemble the physiological loads like when slow walking. However if bone healing takes place before high cycle numbers as those in this study can be reached. Hence one should proceed with caution when applying the results of the present study to clinical settings.

## Conclusions

In summary all the tested plates showed sufficient strength during static loading. All specimens failed due to a fracture of the opposite cortical bone. The TomoFix std showed a higher degree of stability than the small stature version. The results of the cyclic load to failure tests show that the stability of the bone-implant constructs is correlated with the fixation design. The ContourLock plate with its wider T-shaped proximal end showed a higher lifespan prior to failure, followed by the iBalance implants due their closed-wedge design which provides higher stiffness to the bone-implants constructs. The TomoFix and the PEEKPower plates with their narrow T-shaped proximal ends showed less rigidity compared to the ContourLock and the iBalance implants. Since healing rates are reported to be high after TomoFix fixation, which is supposedly due to the callus-massage effect of the implant and the elastic bone-implant construct, it remains to be seen whether constructs with a higher mechanical strength have higher bone healing rates with an equal amount of intraoperative safeness than the TomoFix plate, the current golden standard.
